# AcrAB-TolC efflux pump system plays a role in carbapenem non-susceptibility in *Escherichia coli*

**DOI:** 10.1186/s12866-019-1589-1

**Published:** 2019-09-05

**Authors:** Shiela Chetri, Deepshikha Bhowmik, Deepjyoti Paul, Piyush Pandey, Debadatta Dhar Chanda, Atanu Chakravarty, Debajyoti Bora, Amitabha Bhattacharjee

**Affiliations:** 1 Department of Microbiology, Assam University, Silchar, Assam India; 2 Department of Microbiology, Silchar Medical College and Hospital, Silchar, India; 3 Department of Statistics, Dibrugarh University, Dibrugarh, India

**Keywords:** AcrAB-TolC, CCCP, Real-time PCR, Carbapenems, *Escherichia coli*

## Abstract

**Background:**

Efflux pump mediated antibiotic resistance is an unnoticed and undetected mechanism in clinical microbiology laboratory. RND efflux systems are known for aminoglycoside and tetracycline resistance whereas their role in carbapenem non-susceptibility is not established. The study was undertaken to investigate the role of efflux pump in providing resistance against carbapenems and their response against concentration gradient carbapenem stress on the transcriptional level of the AcrAB gene in the clinical isolates of *Escherichia coli* from a tertiary referral hospital of Northeast India.

**Results:**

Out of 298 non-susceptible *Escherichia coli* isolates 98 isolates were found to have efflux pump mediated carbapenem non-susceptibility. Among them thirty-five were non carbapenemase producers and their expressional levels were verified using qRT-PCR under concentration gradient carbapenem stress. In this study, a strong correlation between ertapenem resistance and AcrA overexpression was observed which has not been reported previously. Further, it was observed that imipenem stress increased AcrB expression in *Escherichia coli* which holds the novelty of this study. Additionally, the transcription of AcrR was insistently increased which is much higher than the transcriptional level of AcrA under concentration gradient carbapenem stress condition.

**Conclusion:**

The study established that AcrAB pump is a relevant antibiotic resistance determinant in bacterial pathogen, has an important role in developing resistance against carbapenem group of antibiotics.

**Electronic supplementary material:**

The online version of this article (10.1186/s12866-019-1589-1) contains supplementary material, which is available to authorized users.

## Background

Efflux pump mediated antibiotic resistance is an important intrinsic mechanism within bacteria which often remained unnoticed and undetected in clinical microbiology laboratory. *Escherichia coli*, one of the most frequently isolated multidrug resistant pathogens possess many efflux pump systems that extrude metabolic waste, toxic substances and antimicrobial agents [1]. There are many published reports on both the intrinsic and acquired resistance mechanisms present in *Escherichia coli* along with the involvement of several elements which contributes to these mechanisms directly or indirectly [2]. However, recently published articles indicate that the characteristic phenotype of non-susceptibility to antibiotics of a given bacterial species depend collective coordination of different element that has been named as intrinsic resistome. The most relevant causes may be lack of target modification activity of chromosomally encoded antibiotic inactivating enzymes or reduced permeability and increased efflux [3–5]. The first efflux pump to be described in *Escherichia coli* was Tet belonging to MFS family which was plasmid encoded [6]. The most important types for the maintenance of *Escherichia coli* in the human gut are AcrAB-TolC, EmrAB-TolC and MdtM for extruding bile salts, mammalian steroids and different antibiotics. AcrAD-TolC system is reported to have role in aminoglycoside resistance although AcrAB-TolC system in tetracycline resistance along with a wide variety of drugs and other compounds of different size and physiochemical properties is well described [7–9]. However, their role in carbapenem resistance is still unknown. Hence, present study was undertaken to assess transcriptional response of AcrAB-TolC and AcrAD-TolC pump against sub-inhibitory concentration gradient stress of carbapenem antibiotics in clinical isolates of *Escherichia coli*.

## Results

**Fig. 1:**
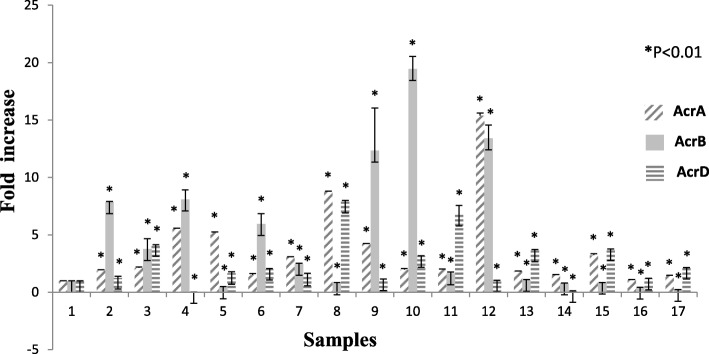
Expression of *AcrA*, *AcrB* and *AcrD* gene of test isolates under normal condition (without stress) relative to *Escherichia coli* ATCC 25922. Footnote: The RQ values mentioned are the average of all the test isolates (representatives of all 16 clusters) when tested in triplicate. 1=Indicates the control used in the study (E. coli ATCC 25922), 2-17=Test isolates, * represents *P* < 0.01

**Fig. 2:**
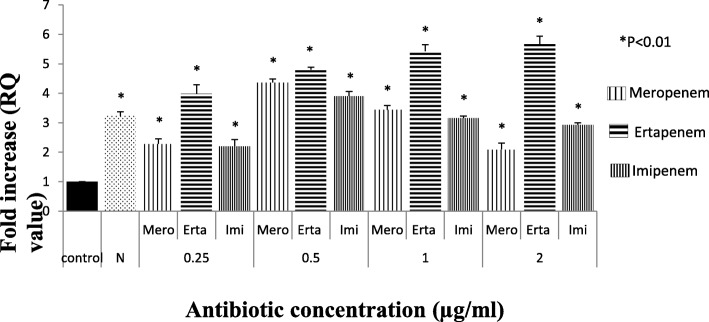
Expression of *AcrA* gene of test isolate under Carbapenem stress relative to *Escherichia coli* ATCC 25922. (The value against each bar is the average RQ value of all the test isolates). Footnote: The RQ values mentioned are the average of all the test isolates (representatives of all 16 clusters) when tested in triplicate. *N* = Isolate in normal condition without stress, C= Control (E. coli ATCC 25922) * represents *P* < 0.01

**Fig. 3:**
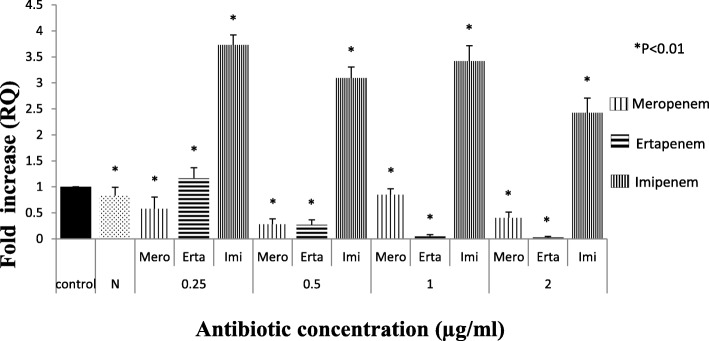
Expression of *AcrB* gene of test isolate under Carbapenem stress relative to *Escherichia coli* ATCC 25922. (The value against each bar is the average RQ value of all the test isolates). Footnote: The RQ values mentioned are the average of all the test isolates (representatives of all 16 clusters) when tested in triplicate. *N* = Isolate in normal condition without stress, C= Control (E. coli ATCC 25922) * represents *P* < 0.01. N- Isolate in normal condition without stress, C- control (E. coli ATCC 25922), * represents *P* < 0.01

**Fig. 4:**
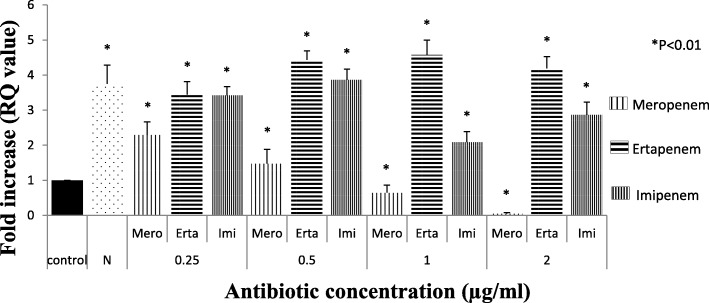
Expression of *AcrD* gene of test isolate under Carbapenem stress relative to *Escherichia coli* ATCC 25922. (The value against each bar is the average RQ value of all the test isolates). Footnote: The RQ values mentioned are the average of all the test isolates (representatives of all 16 clusters) when tested in triplicate. N- Isolate in normal condition without stress, C- control (E. coli ATCC 25922) * represents *P* < 0.01

**Fig. 5:**
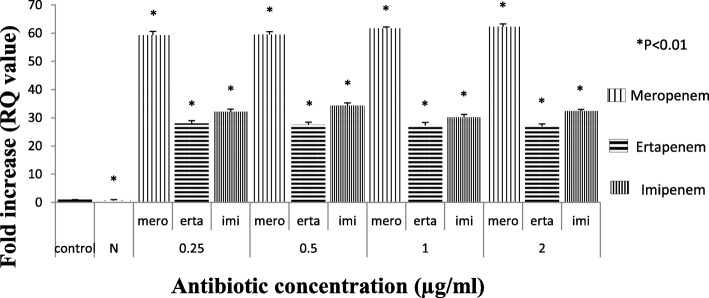
Expression of *AcrR* gene of test isolate under Carbapenem stress relative to *Escherichia coli* ATCC 25922. (The value against each bar is the average RQ value of all the test isolates). Footnote: The RQ values mentioned are the average of all the test isolates (representatives of all 16 clusters) when tested in triplicate. N- Isolate in normal condition without stress, C- control (E. coli ATCC 25922) * represents *P* < 0.01

Among 298 carbapenem non-susceptible *Escherichia coli* isolates, 84 were meropenem resistant. However, 32.9% (98/298) showed efflux pump mediated carbapenem resistance phenotypically when tested against meropenem in the presence of an efflux pump inhibitor with respect to control strains (Additional file 1: Table S1). Sixty-three isolates were producing carbapenemase as observed by Modified Hodge test and carbaNP test among them *bla*_NDM-1_ was detected in 22, *bla*_NDM-7_ was detected in 6, *bla*_OXA-23_ in 17 and *bla*_OXA-48_ in 8 isolates, whereas in 10 isolates PCR experiment could not fetch any amplification although they were modified Hodge test and Carba-NP test positive. Whereas, in CarbaNP and Modified Hodge test negative isolates, carbapenemase genes were not detected using specific primers (NDM, OXA-48, OXA-23, KPC, IMP and IMI/NMC). All the carbapenem non-susceptible and carbapenemase non-producing isolates were selected for further study. The susceptibility testing revealed that most of the isolates (among 35 phenotypically efflux pump positive and carbapenemase negative) were susceptible towards amikacin (65.71%) followed by cefepime (57.14%) and co-trimoxazole (51.42%). MIC result showed majority of the isolates were above the break point against ertapenem (88.5%) which is followed by imipenem (77%) and meropenem (60%) (Table 1) whereas all of them were susceptible to colistin and polymixin B. Based on susceptibility, MIC value and pulse field gel electrophoresis pattern (Additional file 1: Figure S2A, S2B and S2C) 16 groups/clusters were formed and one representative of each group/cluster was further studied for expressional analysis. Over-expression of AcrAB and AcrAD efflux pump was observed in nine isolates and two isolates respectively (Fig. 1). It was observed that the transcriptional expression of AcrA was increased in response to ertapenem stress(*P* <  0.01) whereas against meropenem, the expression of AcrA was elevated upto a particular concentration (0.5 μg/ml) above which a substantial decrease in the expression was noticed. A quite similar response of AcrA was observed against imipenem as well (Fig. 2). In case of AcrB, hyper expression was detected against imipenem stress irrespective of concentrations whereas it was inversely proportional to the increasing concentration of ertapenem (Fig. 3). For AcrD a consistent decrease in the expression under meropenem stress was observed. While against ertapenem exposure the expression was increased upto certain level and beyond that a decline was observed. Against imipenem, the expression of AcrD did not show any specific pattern (Fig. 4). In comparison to *Escherichia coli* ATCC 25922 under normal condition without any stress, a higher expression level of local regulator *acrR* against carbapenem stress was observed in carbapenem resistant isolates. Messenger RNA sequencing of AcrR and AcrS did not show any observable mutation in the sequence (Fig. 5).

**Table 1:** Results of MIC of test isolates against carbapenems (Interpreted as per CLSI guidelines 2017)

Antibiotics	MIC range examined (μg ml^− 1^)														No. (%) of isolates above the breakpoint (*N* = 35)
	< 0.25	0.25	0.5	1	2	4	8	16	32	64	128	256	512	> 512	
Meropenem	12	–	–	2	1	1	1	4	2	6	1	1	–	4	60 (*n* = 21)
Ertapenem	4	–	1	5	1	3	–	–	3	6	3	5	2	3	88.6(*n* = 31)
Imipenem	8	–	1	7	7	–	3	3	3	4	3	2	1	1	77 (*n* = 27)

*N* Total number of isolates, *n* Total number of isolates above the break point

Break point: Ertapenem > 2 μg/ml, imipenem > 4 μg/ml, Meropenem > 4 μg/ml

## Discussion

Dissemination of multi-drug resistant (MDR) bacteria is a major public health concern. Mutations have been found to be responsible for the emergence of the MDR phenotypes and explain how they acquire antibiotic resistance [10, 11]. Multiple mutations are often required to acquire high levels of resistance to a specific drug [12, 13]. However, there are a number of mechanisms evolved by which the bacteria survive against any noxious agents. Among the possible mechanisms for multi-drug resistance, efflux pump systems are membrane proteins which transport toxic substances including antimicrobials out of the cell with over expression of the tripartite pump system resulting in to antibiotic resistance [14]. Efflux pumps such as AcrAB-TolC and MexAB-OprM, are essential for bacterial survival and colonization/virulence, especially during the course of infection when the pathogen is attacked by toxic substances or adhere with the host [15]. The AcrAB-TolC is a constitutive system in *Escherichia coli*, and largely has a role in characteristic intrinsic resistance to antimicrobials such as erythromycin and fusidic acid as well as dyes and detergents [16, 17]. Efflux pump mediated tigecycline resistance in *Klebsiella pneumoniae* was reported from China where in higher expression of efflux pump systems AcrAB-TolC and OqxAB was observed and the expression of AcrB gene was found to be associated with ramA and marA expression [18]. Carbapenems which are considered drugs of last resort has been used quite effectively for decades. The idea about this antibiotic was compromised by the emergence of carbapenem-hydrolysing ß-Lactamase producing strain of *Klebsiella pneumoniae* [19]. Furthermore, the scenario is more complicated in Indian subcontinent after arrival of New Delhi metallo-Beta lactamases in the current decade [20]. However, in a study from India in hospital isolates of *P. aeruginosa* it was observed that MexAB-OprM efflux pump can significantly contribute to meropenem resistance when an acquired resistant mechanism is absent [21].According to the study done by Charleric B*et al* 2003, *Enterobacter aerogenes* of clinical origin exhibited resistance against β-lactam and other group of antibiotics. Efflux pump activity was observed against quinolone, tetracycline and chloramphenicol along with over-expression of AcrA within these imipenem resistant strains [22]. In this study a strong correlation between ertapenem resistance and AcrA over-expression was observed which has not been reported previously. Also, AcrB over-expression against imipenem stress observed in this study is unique of its own. Efflux pump system AcrD, which is a transporter protein belonging to RND super-family of efflux pump known to be responsible for efflux of aminoglycosides [23] did not show any relation with carbapenem resistance in our study. According to work done by Elkins and Nikaido in 2002, for efficient efflux of amphiphilic substrate AcrD requires an association with AcrA in intact cells [24] and probably this protein when combined with AcrA has a role in carbapenem non-susceptibility. Mutations in drug target genes are still assumed to be the primary mechanism of drug resistance. To investigate causes of the increased expression of AcrR and AcrS in AcrAB overexpressed isolates, mRNA sequencing of the regulatory regions was performed and it was confirmed that efflux pump mediated carbapenem resistance does not have any mutational event. In 1996, Dzwokai Ma and co-workers observed that the transcription of AcrAB increased under general stress condition further investigated the role of the local repressor AcrR under general stress condition [24]. Surprisingly, they found the transcription of AcrR was persistently increased by all these conditions, and the extent of increase was even higher than that seen for AcrA though local repressor AcrR is linked as a repressor for AcrAB. In our study in the isolates with increased AcrAB expression under carbapenem stress showed much higher expression of AcrR which is unexpected. In agreement with the earlier study [24] we also hypothesize that stress induced transcription of AcrAB is probably under the control of global transcriptional regulators.

## Conclusion

The current work investigated the role of efflux pump mediated resistance against carbapenems and their response against concentration gradient carbapenem stress on the transcriptional level of the AcrAB gene from the clinical isolates of *Escherichia coli* from a tertiary referral hospital of India. The study established that AcrAB pump is a relevant antibiotic resistance determinant in bacterial pathogen, has a vital role in developing resistance against carbapenem group of antibiotics.

## Methods

### Bacterial isolates

A total of two hundred ninety-eight carbapenem non-susceptible isolates of *Escherichia coli* were obtained from the patients who were admitted to or attended the clinics of Silchar medical college and hospital, India between April 2014 and March 2015. Isolates were selected based on non-susceptibility against at least one of the carbapenems (meropenem, ertapenem and imipenem) tested by disc diffusion method as per CLSI guideline 2017 [25]. All the isolates were identified based on standard biochemical reactions like, catalase, oxidase, IMViC, urease, TSI, nitrate reduction and sugar fermentation etc. [26] and 16S ribosomal DNA sequencing [27].

### Phenotypic detection of efflux pump mediated carbapenem resistance

Detection of efflux pump activity of *Escherichia coli* isolates was performed by using meropenem (10 μg, Himedia, Mumbai, India) with and without an efflux pump inhibitor carbonyl cyanide m –chlorophenylhyrazone (CCCP) (12.5 μM), (Himedia, Mumbai, India). A difference between zone of inhibition of ≥5 mm with the inhibitor and the carbapenem alone confirms to be having efflux pump activity [28]. *E. coli* AG100 and *E. coli* HUE1 (wild type) was used as positive control while *E. coli* AG100A(ΔAcrAB), *E. coli* HUE1(ΔAcrAB-TolC) and *E. coli* ATCC 25922 was used as negative control in the present study. The control strains were obtained from other laboratories (*E. coli* AG100 and *E. coli* AG100A were donated by Prof. Hiroshi Nikaido, University of California, Berkeley USA and *E. coli* HUE1 wild type and *E. coli* HUE1 (ΔAcrAB-TolC) were donated by Prof. Toyotaka Sato, Sapporo Medical University, Japan). Disc with only CCCP (12.5 μM) was also used to rule out any non-specific activity of CCCP.

### Detection of carbapenemases

Modified Hodge test and CarbaNP test was performed to verify the carbapenemase activity in the selected isolates which were phenotypically showing efflux pump activity. Previously confirmed strains of *Escherichia coli* with OXA-48 and NDM-1, NDM-4, NDM-5, NDM-7 were taken as positive control and *Escherichia coli* ATCC25922 as negative control. To validate the absence of carbapenemase genes polymerase chain reaction was carried out in a 96 well thermal cycler (Applied Biosystems) for detection of *bla*_KPC_*, bla*_IMP_, *bla*_VIM_, *bla*_NDM_, *bla*_OXA − 23,_ *bla*_OXA − 48_ and*bla*_OXA − 58_ [29–32].

### Antimicrobial susceptibility testing and minimum inhibitory concentration determination

Disc diffusion method was performed for antimicrobial susceptibility testing of the test isolates and the results were interpreted using CLSI breakpoints. The antibiotics tested includes- ciprofloxacin (5 μg), amikacin (30 μg), cefepime (30 μg), aztreonam (30 μg), ceftriaxone (30 μg), cotrimoxazole (25 μg), ceftazidime (30 μg), levofloxacin (5 μg) gentamicin (10 μg), carbenicillin (10 μg), ceftazidime (30 μg) and Piperacillin/Tazobactam (100/ 10 μg) (Hi-media, Mumbai, India). The minimum inhibitory concentrations (MICs) of carbapenems (meropenem, ertapenem and imipenem), colistin and polymixin B were determined by agar dilution method and the results were interpreted as per CLSI [25] and EUCAST guidelines (Version 9.0) respectively. *Escherichia coli* ATCC 25922 was used as control.

### Strain typing by pulse field gel electrophoresis

All the phenotypically efflux pump positive (carbapenemase non-producing) isolates were typed by pulsed-field gel electrophoresis (PFGE). Agarose blocks were made to prepare DNA and were subsequently digested with *Xba*I (Promega, Madison, WI). The digested DNA fragments were further separated by using CHEF-DR III PFGE system (Bio-Rad, Hercules, CA) for 24 h at 4 V/cm with pulses at 120° angle in a 10 s to 40 s pulse time.

### Determination of transcriptional expression of acrA, acrB and acrD by quantitative real time PCR

Total cellular RNA was isolated from *Escherichia coli* isolates using the QIAGEN Rneasy Mini Kit (QIAGEN, Germany) according to the manufacturer’s recommendation. For cDNA synthesis, QIAGEN Reverse Transcription Kit (QIAGEN, Germany) was used. Real Time PCR was performed for quantification of transcriptional expression using power Sybrgreen PCR master mix reagents kit (Applied Biosystems, Austin, USA) and the expression levels of *acrA*, *acrB* and *acrD* genes were assessed using StepOnePlus quantitative Real Time-PCR (Applied Biosystems, USA) using oligonucleotide primers[acrA(F): 5’CTCTCAGGCAGCTTAGCCCTAA3’, acrA(R): 5’TGCAGAGGTTCAGTTTTGACTGTT3’] [33], [acrB(F): 5’AGCTTCCTGATGGTTGTCGG3’, acrB(R): 5’ACGGCTGATGGCATCTTTCA3’], [acrD(F): 5’GCCGTGCAGCAAGTACAAAA3’, acrD(R): 5’GTATCGCCGGTTTTACGCAC3’]**.** In the PCR reaction, a housekeeping gene (*RpslE*) was used as internal control. Relative quantification was determined by∆∆Ct method as per the software provided by the manufacturer. Each sample was processed in triplicate.

### Transcriptional response of acrA, acrB and acrD against concentration gradient carbapenem stress

Transcriptional levels were also determined after exposing the isolates at different sub-inhibitory concentration of meropenem (Astra Zeneca Limited, India), ertapenem (MSD Pharmaceuticals Pvt. Ltd., India) and imipenem (Hetero Labs Limited, India) ranging from 0.25 μg/ml upto 2 μg/ml by quantitative Real Time PCR. Each sample was processed in triplicates and their relative expression was compared with that of *Escherichia coli* ATCC 25922 (expressed without antibiotic stress).

### Sequencing of acrR and acrS

The isolates with over- expressed efflux pump activity were subjected to cDNA preparation from mRNA using QuantiTect Reverse Transcription Kit (QIAGEN, India). Using Specific primers, [AcrR(F): 5’ATGGCACGAAAAACC3’, AcrR (R): 5’TGCCACTAACGAATAA3’AcrS(F): 5’AAGAACCAAAGCCGAAGCTC3’, AcrS(R): 5’ACATGACACTTAATTCATTCG3’]. The products were amplified by PCR and were sequenced by Sanger’s method. The resulting DNA sequences were analysed using the basic local alignment search tool (http://www.ncbi.nih.gov/BLAST).

### Determination of transcriptional expression of the local regulator acrR gene

Isolates over-expressing AcrAB and AcrAD efflux pump systems were selected and the transcriptional expression of *acrR* were demonstrated by quantitative Real Time PCR using primers (forward primer: 5′ACAAGAAGCGCAAGAAACGC3′ and reverse primer: 5′CCAGCGAGGTGGATGATACC3′). *Escherichia coli* ATCC 25922 was used as a reference strain. Transcriptional response of *acrR* against concentration gradient carbapenem stress was also analysed by Real time PCR assay.

### Statistical analysis

The differences between samples (control, and test isolates with and without stress) were determined with the help of one-way ANOVA followed by Tukey-Kramer (Tukey’s W) multiple comparison test. Differences were considered statistically significant at both 5 and 1% level when *p* <  0.05. SPSS version 17.0 was used for statistical analysis.

## Additional file

Additional file 1: **Figure S2A.** Pulse field gel electrophoresis pattern of carbapenem resistant carbapenemase non-producing isolates. **Figure S2B.** Pulse field gel electrophoresis pattern of carbapenem resistant carbapenemase non producing isolates. **Figure S2C.** Pulse field gel electrophoresis pattern of carbapenem resistant carbapenemase non-producing isolates. **Table S1.** Detailed zone of inhibition towards meropenem alone (as per CLSI guideline 2017) as well as with inhibitor. (DOCX 3007 kb)

## Data Availability

All the data generated in this research work are presented in this research article. In case of any additional information requirement corresponding author will be providing the necessary information as per ethical guidelines.

## Abbreviations

BLAST: Basic local alignment search tool
CCCP: Carbonyl cyanide m -chlorophenylhyrazone
cDNA: Complementary Deoxyribonucleic Acid
CLSI: Clinical laboratory standard Institute
MDR: Multidrug Resistant
MFS: Major facilitator superfamily
MIC: Minimum inhibitory concentration
mRNA: Messenger Ribonucleic Acid
qRT-PCR: Quantitative Real Time Polymerase Chain Reaction
RND: Resistance nodulation division
