# Supplementary material for: AcrAB-TolC efflux pump system plays a role in carbapenem non-susceptibility in Escherichia coli
Source: BMC Microbiol. 2019 Sep 5;19:210. doi: 10.1186/s12866-019-1589-1 (PMC6727511; doi:10.1186/s12866-019-1589-1)
Supplement: Supplementary file 1 — Figure S2A. Pulse field gel electrophoresis pattern of carbapenem resistant carbapenemase non-producing isolates. Figure S2B. Pulse field gel electrophoresis pattern of carbapenem resistant carbapenemase non producing isolates. Figure S2C. Pulse field gel electrophoresis pattern of carbapenem resistant carbapenemase non-producing isolates. Table S1. Detailed zone of inhibition towards meropenem alone (as per CLSI guideline 2017) as well as with inhibitor. (DOCX 3007 kb) [file 12866_2019_1589_MOESM1_ESM.docx]

1 2 3 4 5 6 7 8 9 10 11 12 13 14 15


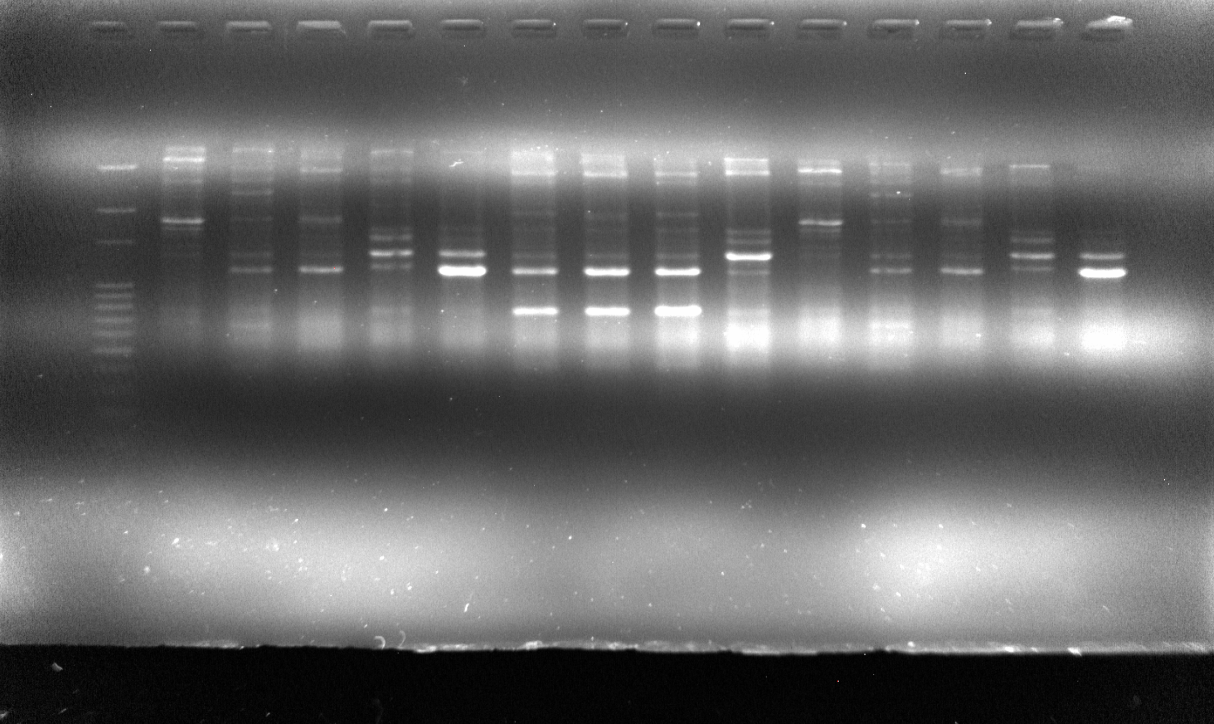


**Figure S2A. Pulse field gel electrophoresis pattern of carbapenem resistant carbapenemase non-producing isolates.**

Lane 1: Ladder, Lane 2: Pulsotype 1, Lane 3 and 12: Pulsotype 2, Lane 4 and 13: Pulsotype 3, Lane 5 and 14: Pulsotype 4, Lane 6: Pulsotype 5, Lane 7,8 and 9: Pulsotype 6, Lane 10: Pulsotype 7, Lane 11: Pulsotype 8, Lane 15: Pulsotype 9.

1 2 3 4 5 6 7 8 9 10 11 12 13 14


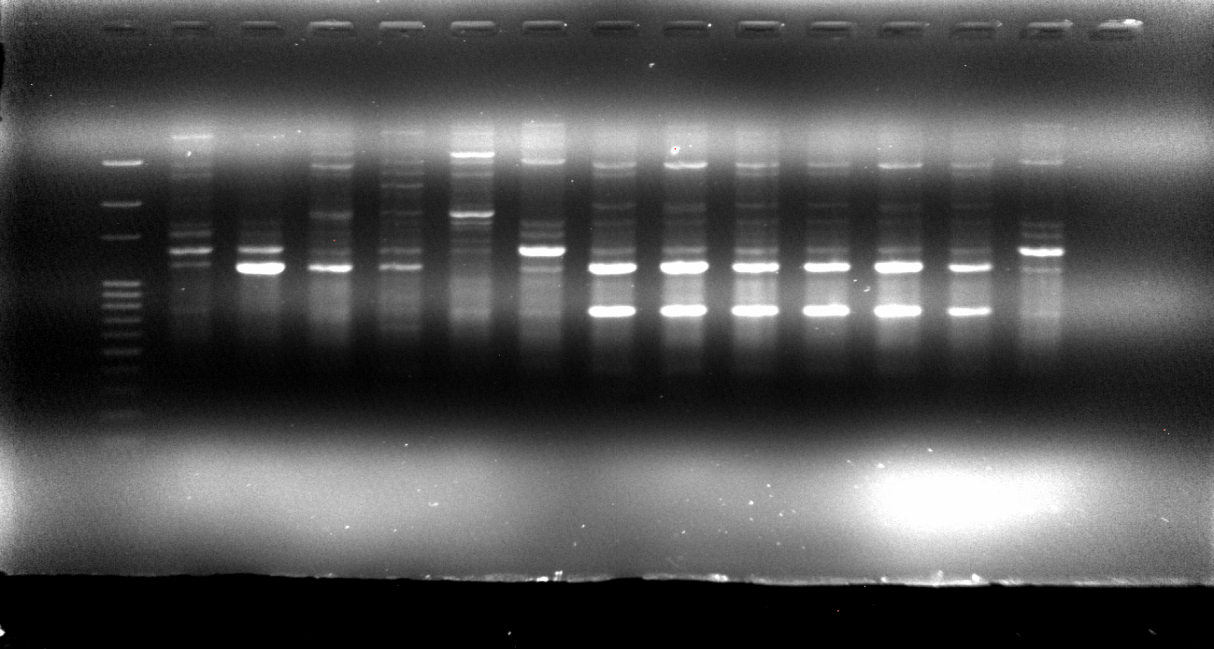


**Figure S2B. Pulse field gel electrophoresis pattern of carbapenem resistant carbapenemase non producing isolates.**

Lane 1: Ladder, Lane 2: Pulsotype 10, Lane 3: Pulsotype 5, Lane 4: Pulsotype 11, Lane 5: Pulsotype 12, Lane 6: Pulsotype 13, Lane 7: Pulsotype 14, Lane 8 to lane 13: Pulsotype 15, Lane: Pulsotype 16

1

9

8

7

6

5

4

3

2


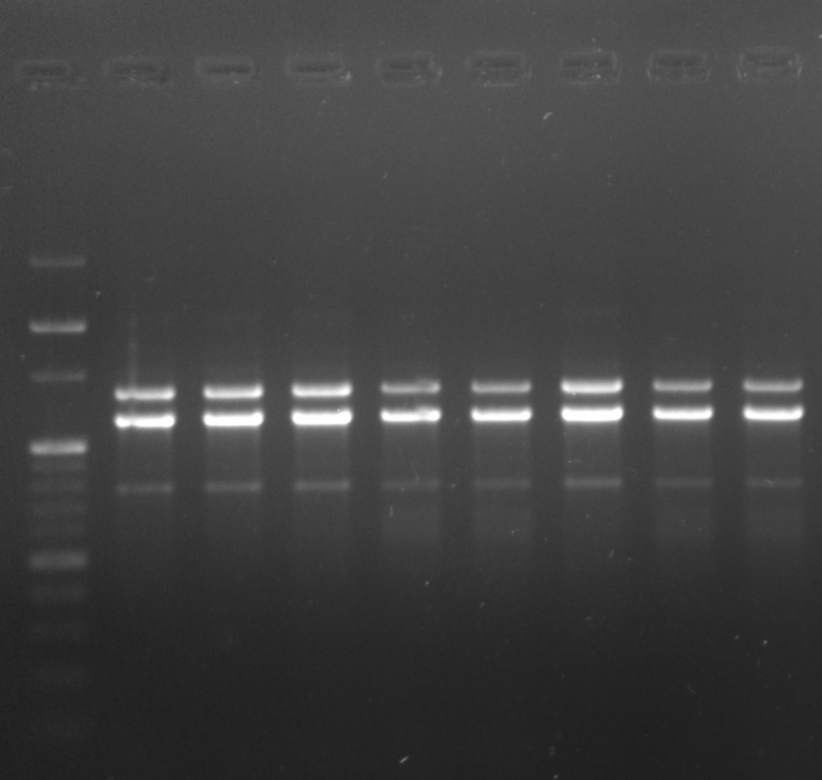


**Figure S2C. Pulse field gel electrophoresis pattern of carbapenem resistant carbapenemase non-producing isolates.**

Lane 1: Ladder, Lane 2 to 9: Pulsotype 5

**Supplementary Table 1:** Detailed zone of inhibition towards meropenem alone (as per CLSI guideline 2017) as well as with inhibitor

| **S. No** | **Sample ID** | **Zone of inhibition (in mm)** | | **Carbapenemase activity (MHT positive)/ carbapenemase genes** |
| --- | --- | --- | --- | --- |
|  |  | **Meropenem alone** | **Meropenem + CCCP (Inhibitor)** |  |
|  | AG100 (Positive control) | 12mm | 23mm |  |
|  | *E. coli* HUE1 wild type (Positive control) | 18mm | 25mm |  |
|  | AG100A (Negative control) | 13mm | 15mm |  |
|  | *E. coli* HUE1(Negative control) | 18mm | 20mm |  |
|  | *E. coli* ATCC 25922 (Negative control) | 30mm | 30mm |  |
|  | SC1 | 24mm | 30mm |  |
|  | SC2 | 20mm | 26mm |  |
|  | SC3 | 15mm | 25mm |  |
|  | SC4 | 20mm | 26mm |  |
|  | SC6 | 24mm | 31mm |  |
|  | SC7 | 9mm | 15mm | +, *bla*_NDM-1_ |
|  | SC9 | 20mm | 27mm |  |
|  | SC11 | 20mm | 29mm | +, *bla*_OXA-23_ |
|  | SC12 | 20mm | 25mm |  |
|  | SC14 | 10mm | 24mm | +, *bla*_NDM-1_ |
|  | SC17 | 20mm | 27mm | +, *bla*_OXA-23_ |
|  | SC18 | 12mm | 19mm | +, *bla*_NDM-7_ |
|  | SC20 | 24mm | 29mm |  |
|  | SC23 | 14mm | 19mm |  |
|  | SC25 | 12mm | 20mm | +, *bla*_NDM-1_ |
|  | SC28 | 11mm | 18mm | +, *bla*_OXA-48_ |
|  | SC35 | 11mm | 20mm | +, *bla*_OXA-23_ |
|  | SC38 | 8mm | 14mm | +, *bla*_NDM-1_ |
|  | SC39 | 18mm | 24mm | +, *bla*_OXA-48_ |
|  | SC40 | 7mm | 17mm | +, *bla*_NDM-1_ |
|  | SC43 | 19mm | 24mm | + |
|  | SC46 | 19mm | 24mm | +, *bla*_OXA-23_ |
|  | SC49 | 20mm | 25mm |  |
|  | SC51 | 19mm | 27mm | +, *bla*_NDM-1_ |
|  | SC52 | 17mm | 22mm | + |
|  | SC53 | 16mm | 23mm | +, *bla*_OXA-23_ |
|  | SC55 | 8mm | 18mm | +, *bla*_NDM-1_ |
|  | SC 59 | 19mm | 25mm | + |
|  | SC62 | 16mm | 21mm | +, *bla*_OXA-23_ |
|  | SC65 | 14mm | 21mm | +, *bla*_OXA-23_ |
|  | SC68 | 17mm | 23mm |  |
|  | SC70 | 16mm | 22mm |  |
|  | SC73 | 16mm | 23mm |  |
|  | SC77 | 16mm | 27mm | +, *bla*_OXA-48_ |
|  | SC79 | No Zone | 12mm | +, *bla*_NDM-1_ |
|  | SC80 | 10mm | 22mm | +, *bla*_OXA-48_ |
|  | SC81 | 16mm | 25mm | + |
|  | SC82 | 19mm | 26mm |  |
|  | SC84 | 16mm | 25mm | +, *bla*_OXA-23_ |
|  | SC85 | 16mm | 28mm | +, *bla*_NDM-7_ |
|  | SC89 | 30mm | 35mm |  |
|  | SC91 | 12mm | 19mm |  |
|  | SC92 | No zone | 12mm | +, *bla*_NDM-1_ |
|  | SC94 | No zone | 11mm | +, *bla*_NDM-1_ |
|  | SC96 | 10mm | 19mm |  |
|  | SC98 | 12mm | 20mm | +, *bla*_OXA-48_ |
|  | SC99 | 13mm | 22mm | +, *bla*_OXA-23_ |
|  | SC100 | 10mm | 22mm | +, *bla*_NDM-1_ |
|  | SC104 | 16mm | 25mm | + |
|  | SC105 | 19mm | 28mm | + |
|  | SC106 | 16mm | 25mm | +, *bla*_NDM-1_ |
|  | SC107 | 16mm | 20mm |  |
|  | SC110 | 30mm | 35mm |  |
|  | SC111 | 17mm | 26mm | +, *bla*_OXA-23_ |
|  | SC112 | 20mm | 26mm |  |
|  | SC113 | No zone | 15mm | +, *bla*_NDM-1_ |
|  | SC115 | No zone | 16mm | +, *bla*_NDM-1_ |
|  | SC116 | No zone | 17mm | +, *bla*_NDM-1_ |
|  | SC119 | 13mm | 21mm | + |
|  | SC122 | 16mm | 21mm | +, *bla*_OXA-23_ |
|  | SC125 | 15mm | 22mm | + |
|  | SC129 | No zone | 15mm | +, *bla*_OXA-23_ |
|  | SC130 | No zone | 14.5mm | +, *bla*_OXA-23_ |
|  | SC131 | No zone | 19.5mm | +, *bla*_OXA-23_ |
|  | SC132 | 11mm | 16.5mm |  |
|  | SC133 | 17mm | 21mm |  |
|  | SC134 | 20mm | 26mm |  |
|  | SC139 | No zone | 15mm | + |
|  | SC140 | No zone | 16mm | +, *bla*_OXA-48_ |
|  | SC144 | No zone | 17mm | +, *bla*_OXA-23_ |
|  | SC145 | 13mm | 21mm | +, *bla*_NDM-1_ |
|  | SC146 | 16mm | 21mm |  |
|  | SC147 | 15mm | 23mm | +, *bla*_NDM-1_ |
|  | SC148 | 10.5mm | 16mm |  |
|  | SC150 | 12mm | 20mm | +, *bla*_OXA-48_ |
|  | SC152 | No zone | 15mm | +, *bla*_NDM-1_ |
|  | SC156 | 12mm | 17.5mm |  |
|  | SC158 | 11mm | 18mm | +, *bla*_OXA-23_ |
|  | SC160 | 10mm | 19mm |  |
|  | SC162 | No zone | 15mm | +, *bla*_OXA-48_ |
|  | SC165 | 9.5mm | 14.5mm | +, *bla*_NDM-1_ |
|  | SC166 | 11mm | 17mm |  |
|  | SC167 | 10mm | 16mm |  |
|  | SC168 | 7mm | 19mm | +, *bla*_NDM-7_ |
|  | SC169 | 8mm | 13mm | +, *bla*_OXA-23_ |
|  | SC171 | 7.5mm | 14mm | +, *bla*_NDM-1_ |
|  | SC172 | 9mm | 14.5mm |  |
|  | SC175 | 12mm | 18mm |  |
|  | SC179 | 11mm | 17.5mm |  |
|  | SC183 | 12mm | 22mm | +, *bla*_NDM-1_ |
|  | SC186 | 13.5mm | 18.5mm |  |
|  | SC189 | 12mm | 17mm |  |
|  | SC190 | 14.5mm | 26mm |  |
|  | SC191 | 9mm | 16mm | +, *bla*_NDM-7_ |
|  | SC192 | 9mm | 15mm | + |
|  | SC193 | No zone | 13mm | +, *bla*_NDM-7_ |
|  | SC194 | No zone | 13mm | +, *bla*_NDM-7_ |
|  | SC196 | 14mm | 22mm | +, *bla*_NDM-1_ |

Footnote: 84 test isolates were meropenem resistant as per CLSI guideline 2017.
